# Effect of partner presence on emotion regulation during parent–child interactions

**DOI:** 10.1038/s41598-024-60998-4

**Published:** 2024-05-22

**Authors:** Yael Enav, Marguerite Knudtson, Amit Goldenberg, James J. Gross

**Affiliations:** 1 Department of Counseling and Human Development, University of Haifa, Haifa, Israel; 2 Stanford University, Stanford, USA; 3 Harvard Business School, Boston, USA

**Keywords:** Psychology, Human behaviour

## Abstract

Having people around, especially if they provide social support, often leads to positive outcomes both physically and mentally. Mere social presence is especially beneficial when it comes from a loved one or romantic partner. In these studies, we aim to expand the understanding of how the presence of one’s romantic partner affects emotion regulation in parental situations. Specifically, we examined how partner presence influences the parent’s emotional intensity, emotion regulation, and interpretation of their child’s emotion regulation. We examined these questions in parents of both non-autistic children (Study 1) as well as autistic children (Study 2), which we hypothesize leads to more intense emotional interactions. The parents of autistic children were better able to regulate their emotions when their partners were present compared to when they were absent. Furthermore, in both studies, parents’ ratings of their children’s ability to regulate their emotions were higher when their parent’s partner was present compared to when the partner was absent. However, in both studies, we found no significant difference in the parents’ emotional intensity when their partners were present compared to when their partners were absent during the emotionally charged interaction with their child. Our findings help highlight the impact of partner presence on parent and child emotion regulation.

## Introduction

Imagine that your child is having a tantrum because she does not like what you prepared for dinner, even though it is exactly what she asked for. You are exhausted after a long day at work and frustrated with her behavior. You take a deep breath and try to calmly handle the situation. Now imagine the same scenario, but this time with your partner present. What—if anything—is different? Does the presence of your partner have a calming effect on you? Or does his or her presence lead you to get even angrier? And what would be the effect of the presence of your partner on your child? The studies presented in this paper investigate how the presence of one’s partner affects emotions and emotion regulation during emotionally charged interactions with one’s child. The researchers examined how partner presence influences the intensity of parents’ emotions, their emotion regulation skills, and their interpretations of their children’s emotion regulation. The authors examine these questions both in parents of non-autistic children as well as in parents of autistic children, which the authors hypothesize leads to more intense emotional interactions.

### The presence of others and emotion regulation

People are highly sensitive to the presence of others around them. Generally, the presence of others, especially if they provide social support such as positive interactions, affirmation, and emotional aid, leads to positive physical and mental outcomes^1–6^. Social presence is especially beneficial when it comes from a loved one^7^. This tendency is seen in a very early age, when the mere presence of a parent helps infants regulate their own emotions and manage psychological stress^7,8^. Hofer^9^ proposed that what begins as the regulation of basic physiological needs gradually transforms into the regulation of emotion in adulthood. Emotion regulation is the individual’s ability to influence his or her subjective experience and expression of emotions^10^. Studies examining emotion regulation between adults have shown that social support both reduces negative affect^2,5^ and increases positive affect^11^. In some social situations, merely the presence of another individual promotes emotion regulation^12,13^.

### Emotion regulation in romantic relationships

Romantic relationships have a positive influence on emotions and emotion regulation^14^. For example, Coan et al.^15^ found that women exposed to a mild shock reported less pain when holding another person’s hand but greater regulation occurred if a woman was holding her spouse’s hand. In many cases emotion regulation may occur without partners’ explicit motivation to regulate their partner’s emotion. Emotional interactions between romantic partners often function as emotion regulation in which the individuals influence each other’s emotions towards a stable state^16–18^. Emotional interactions between romantic partners refer to the ways in which individuals express and exchange emotions within the context of their romantic relationship. These interactions encompass a wide range of activities, including trusting the partner, fulfilling their needs, and promoting relationship cohesion^14^. An example of emotional interactions between romantic partners that function as emotion regulation could be when one partner expresses sadness about a challenging day at work and the other partner responds with empathy and support. We observe that this phenomenon is prevalent among securely attached couples, characterized by their confidence in the romantic relationship. Such couples typically demonstrate greater marital satisfaction and better psychological well-being, which contribute to enhanced emotion regulation compared to distressed couples, who lack security in their romantic relationship^19^. This underscores the correlation between the quality of a romantic relationship and emotion regulation.

Despite the evidence that partner presence contributes to a reduction in negative emotions and emphasizes enhanced emotion regulation^19^, some studies bring to light a more complex nature of emotion regulation between romantic partners. Goldenberg et al.^20^ discovered that romantic partners often engage in emotion regulation to counterbalance their partner’s emotional reactions. These processes, termed "emotional compensation," primarily occur in situations where there is high certainty regarding the anticipated response. For instance, if one’s child stole money from a friend, and one’s partner downplayed the severity of the situation, one may intensify his or her own anger to ensure that one’s child comprehends the gravity of the situation^20^.

The influence of a romantic partner on the emotion regulation of the other partner is a complex and under-studied phenomenon. Recognizing this gap, our study aims to provide further insight into this dynamic and unravel the impact of partner presence on emotion regulation during parent–child interactions. Given that a romantic partner influences his or her partner’s emotion regulation^19^ we sought to investigate whether partner presence influences emotion regulation in challenging emotional situations involving children. Furthermore, considering that parenting a child with autism typically involves heightened stress compared to parenting a typically developing child^21^, we extended our inquiry to include parents of children with autism after initially examining this question with parents of typically developing children. Through this comprehensive approach, we aim to deepen our understanding of the intricate interplay between romantic partner presence and emotion regulation in the context of parenting.

### Emotion regulation in parent–child dyads

The cultivation of emotion regulation is a pivotal aspect of a child’s social development^22,23^. The characteristics of the parent–child bond, including emotional warmth, attentiveness, consistent nurturing, and appropriate reactive measures, are closely linked to the development of a child’s ability to manage emotions^24^. Secure attachments between parents and children lay the foundation for children to learn emotion regulation skills^22,25,26^. Notably, the skillset for regulating emotions in children has been associated with a parent’s emotional understanding, engagement, and encouragement^22^.

Furthermore, the way parents handle their emotions and their parenting approach can significantly impact their child’s emotional regulation capacities^27,28^. The way in which parents express emotions serves as a critical example for the child in learning emotional expression. Parents who struggle with recognizing and articulating their feelings may likewise face challenges in acknowledging and addressing their child’s emotional needs, which is particularly pertinent when it comes to responding to children with psychiatric conditions^29^. For instance, misinterpretation of a child’s emotions by parents could lead to responses that are inaptly emotional and could exacerbate the situation^30^.

While all parents encounter stressful situations with their children, parents of children with developmental disabilities, particularly autism, report higher levels of stress and other negative affective states than parents of non-autistic children^31,32^. One possible reason for this heightened stress could be the challenges children with autism spectrum disorder (ASD) face in regulating their emotions. A recent meta-analysis underscores the prevalence of emotion regulation difficulties among children and adolescents with ASD compared to non-autistic controls^33^.

### The present research

As parents’ emotion regulation is an important resource in managing high stress and negative affective states^34^, our studies aim to examine emotion and emotion regulation during parent–child interactions in both families with typically developing children and families of children with autism. Based on prior literature, it is known that parents have a significant influence on their children’s emotion regulation abilities^26^, but it is unknown if there is an influence on child emotion regulation associated with the presence of both parents during parent–child interactions.

The goal of the present research is to examine the effect of the presence of parents’ partners on the intensity of parents’ emotions and their (and their child’s) ability to regulate their emotions during a naturally occurring, emotionally charged interaction with their child. We compared two situations: one in which the partner was present during the interaction and one in which the partner was absent. We hypothesized that when the partner was present, parents would report (1) a lower intensity of emotions; (2) a greater ability to regulate their emotions; (3) and an increase in their children’s abilities to regulate their emotions. We ran two correlational studies to test these hypotheses.

## Study 1: The effects of the partner’s presence with parents of typically developing children

### Methods

#### Participants

Three hundred sixty nine participants were recruited using the online platform MTurk, a crowdsourcing marketplace that enables users to engage in survey completion for financial compensation. To ensure data quality, MTurk tracks and shares users’ number of approved hits (the number of MTurk tasks the user has completed) as well as their approval rate (the percent of completed tasks that the MTurk requesters approved). All of the participants in this study were Americans with at least 100 approved hits and an approval rate of about 95%. We monitored participants’ IP addresses to ensure that they were not repeating the study. We also performed reading checks, reviewing the text descriptions of the emotionally charged incidents, to monitor the contents of the text inputs produced by participants and ensure that the study was not being completed by bots. Participants received 260 cents per 20 min of participation with a goal of reaching a rate of $8 an hour.

Forty seven participants were omitted from our analyses. Of these, 28 incorrectly responded to the reading check and 19 gave meaningless responses in the text entry. A final sample of 322 participants (62.73% male, 37.26% female, age 21–62, M = 36.15, SD = 8.05) was included in the analyses. All participants indicated that they had a child under the age of 18 (M age = 8.91, SD = 5.41). The majority of participants indicated that they were married (80.43%), 13.66% were single, and 5.59% were divorced. 73.91% of participants were White, 8.39% were Black, 6.83% were Latino/a, 5.59% were of Asian descent, and 4.66% indicated they were of another race (see Table 1).

**Table 1:** Study 1 demographics for study 1.

Demographic factor		Frequency	Percent
Gender	Male	232	62.73
	Female	137	37.26
Race	White	274	73.91
	Black	31	8.39
	Latino/a	26	6.83
	Asian	21	5.59
	Other	17	4.66
Marital status	Married	297	80.43
	Single	51	13.66
	Divorced	21	5.59
Parents’ age (yrs)	Range	Mean	SD
	21–62	36.15	8.05

Gender: distribution of participants by gender.

Race: distribution of participants by race.

Marital status: distribution of participants by marital status.

Parents’ age (years): distribution of parents’ age, including range, mean, and standard deviation.

SD, standard deviation.

#### Procedure

Participants read the prompt “Please describe an emotionally charged interaction you had with your child in the last week” and responded using a text entry form. They were then asked four questions about the interaction they described, which they answered using a Likert scale ranging from 1 (Not at all) to 5 (Very). The first question measured the intensity of the parent’s emotions during the interaction (“How intense were your emotions during this interaction?”), the second question was used to measure the parent’s emotion regulation (“How well did you deal with your emotions?”), and the third question measured the parent’s assessment of their child’s emotion regulation during the interaction (“How well did your child handle their emotions at that level of intensity?”). Finally, participants indicated whether their partner was present during the interaction and, if so, whether the partner was involved in addition to the perceived intensity of the partner’s emotions.

To examine the impact of the partner’s presence on parents’ emotional intensity, parental emotion regulation, and parents’ interpretation of their children’s emotion regulation, a linear regression was conducted. This predicted parental emotional intensity, parental emotion regulation and child emotion regulation based on the partner’s presence. As there was a significant difference between groups for the demographic factor of parents’ sex and parents’ sex predicted differences in all three outcome variables, we used parents’ sex as a covariate during analyses. However, this did not change the significance of the results. Analyses were limited to data that were collected and no method was used to fill in missing data. The experimental protocol was approved by Stanford University institutional committee. All methods were performed in accordance with the relevant guidelines and regulations.

#### Data availability

All data generated or analyzed during this study are included in this published article and its supplementary information files.

#### Results

Based on participants’ responses to whether their partner was present during the emotionally charged interaction with their child, they were either placed in the partner present group (N = 145) or the partner absent group (N = 201). No significant differences were found between groups for the demographic factors of parents’ age, marital status, employment status, and education level. There was a significant difference between groups for the demographic factor of parents’ sex (partner present group: 52% male, 48% female; partner absent group: 69% male, 31% female) which was controlled for during analyses.

**Figure 1:**
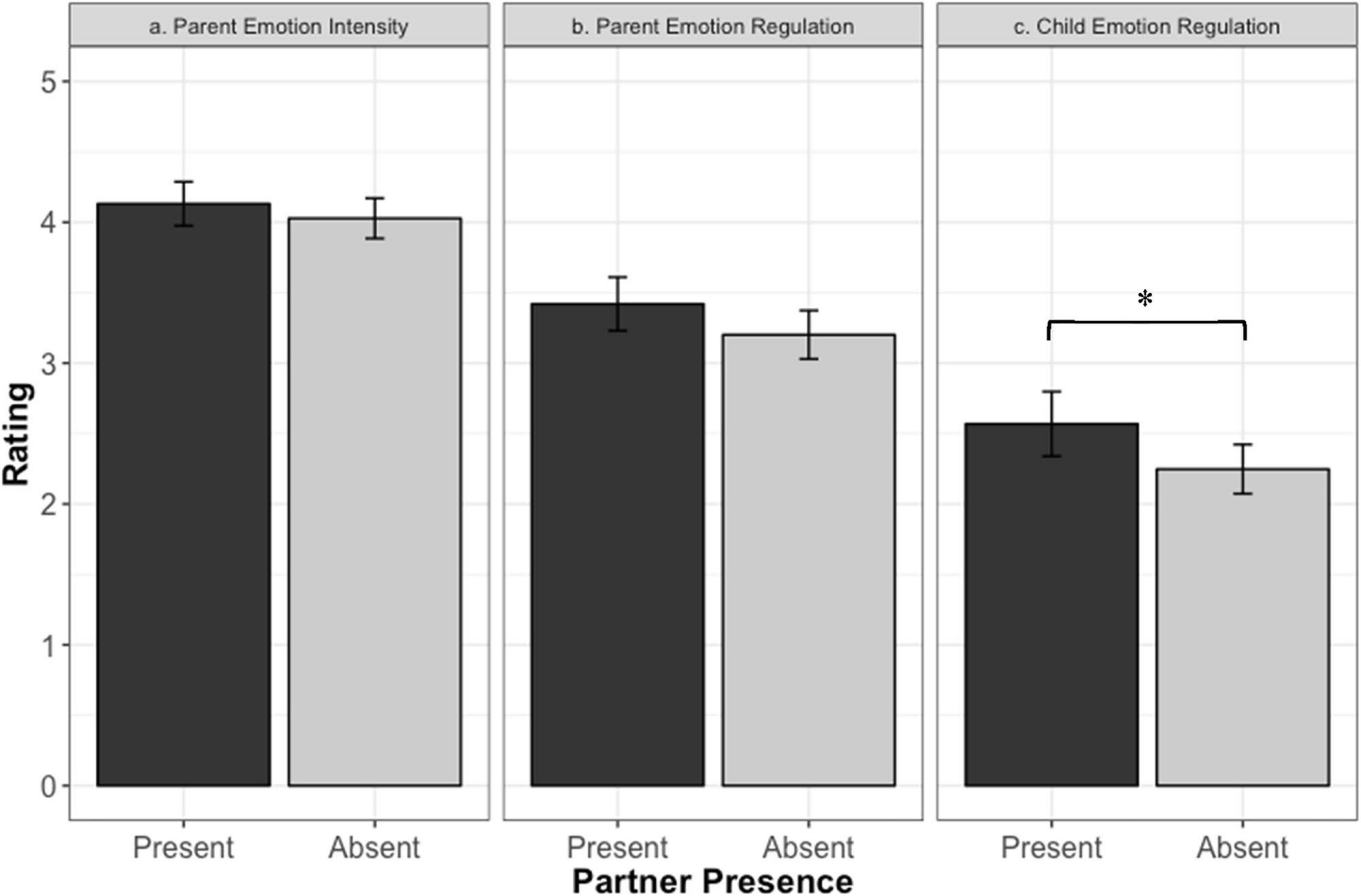
Parents’ ratings of their own and their child’s reactions to the emotional charged interaction with and without their partner present in Study 1. *Note* (**a**) Parents’ ratings of the intensity of their own emotions with and without their partner present (**b**) Parents’ ratings of their own emotion regulation with and without their partner present (**c**) Parents’ ratings of their child’s emotion regulation with and without their partner present. Error bars represent 95% confidence interval.

Results suggested that there was no significant difference in the parents’ emotional intensity when their partner was present (M = 4.13; SD = 0.89) compared to when their partner was absent during the emotionally charged interaction with their child (M = 4.03; SD = 0.97; b = − 0.10 [− 0.32, 0.11]; SE = 0.1095; t(307) = − 0.947; *p* = 0.344, Fig. 1a). Similarly, we found that there was no significant difference in the parents’ ability to regulate their emotions when their partner was present (M = 3.42; SD = 1.08) compared to when their partner was absent (M = 3.21; SD = 1.18; b = − 0.2187 [− 0.48, 0.044]; SE = 0.1336; t(307) = − 1.637; *p* = 0.103, Fig. 1b). Finally, results suggested that participants evaluated their children as being better able to regulate their emotions when their parent’s partner was present (M = 2.57; SD = 1.31) compared to when the partner was absent during the emotionally charged interactions (M = 2.25; SD = 1.20; b = − 0.3214 [− 0.607, − 0.035]; SE = 0.1453; t(307) = − 2.212; *p* = 0.028, d = 0.29, Fig. 1c).

Adding the number of children in the families as a covariate did not impact the significance of the results. For cases in which the partner was present during the emotionally charged incident between parent and child, 54% had the partner involved in the incident while, in the remaining 46%, the partner observed the incident but was not involved. In this subset where partners were present, the involvement of the partner had no significant effect on any of the three outcome variables: parents’ emotional intensity, parents’ ability to regulate their emotions, and children’s ability to regulate their emotions.

## Study 2: The effects of the partner’s presence with parents of autistic children

The aim of the second study was to investigate the same relationships examined in the first study but in parents of autistic children. As described earlier, parents of children with autism are under greater parental stress than parents of typically developing children and their children have greater emotion regulation difficulties. We asked parents to think of an emotionally charged interaction they recently had with their autistic child and then answer similar questions as those in the first study using the same Likert scale. We posited that parents would perceive their children as exhibiting enhanced emotional regulation abilities in the presence of their partners. Moreover, considering the amplified stress levels experienced by parents of children with autism, coupled with their children’s heightened challenges in emotion regulation, we extended our hypothesis to propose that the presence of partners would also aid parents in regulating their own emotions. This supposition is supported by existing research indicating that the presence of a romantic partner during stressful circumstances can facilitate emotion regulation^19,27^.

### Methods

#### Participants

Participants were recruited through the distribution of fliers at clinics, schools, autism awareness events, and by word of mouth. Eighty parents of autistic children and adolescents signed an informed consent form and completed screening procedures. Of these, twelve parents did not complete the study due to time limitations. Sixty-eight participants were included in the final sample.

Participants ranged from 30 to 64 years of age and were primarily female (81.2%). The number of children in each family ranged from 1 to 8 with 2 being the mode. The ages of children ranged from 3 to 18 years and the mean age was 9.93 years. 50% of participants identified as Caucasian, 30.88% identified as Asian or Pacific Islander, 5.88% identified as Latino/a, 10.29% indicated they were of another race, and 2.94% didn’t respond (see Table 2).

**Table 2:** Study 2 demographics for study 2.

Demographic factor		Frequency	Percent
Gender	Male	13	18.8
	Female	55	81.2
Race	Caucasian	34	50
	Asian/Pacific Islander	21	30.88
	Hispanic/Latino	4	5.88
	Other	7	10.29
	Missing	2	2.94
Parents’ age (yrs)	Range	Mean	SD
	30–63	44.70	7.45

Gender: distribution of participants by gender.

Race: distribution of participants by race.

Marital status: distribution of participants by marital status.

Parents’ age (years): distribution of parents’ age, including range, mean, and standard deviation.

SD, standard deviation.

#### Procedure

After expressing interest in participating in the study parents were screened over the phone for their availability, confirmation of their child’s diagnosis, and English proficiency. Following the screening, parents who were deemed as eligible participants were invited to sign the consent form. A questionnaire was given to the parents three times per week for 4 weeks instructing parents to “Please think of an emotionally charged interaction you had with your child with autism in the last 2–3 days.” Parents were then instructed to answer four questions about the interaction using the same Likert scale as in Study 1. Lastly, participants indicated whether their partner was present during the interaction. Participants were required to complete at least two of these four weekly questionnaires to be included in the study. The experimental protocol was approved by Stanford University institutional committee. All methods were performed in accordance with the relevant guidelines and regulations.

#### Data availability

All data generated or analyzed during this study are included in this published article and its supplementary information files.

#### Results

In a similar analysis structure to the one used in Study 1, we compared participants’ responses based on whether the emotionally charged interaction they reported occurred in the presence of their partner (partner present group: N = 188; partner absent group: N = 118). No significant differences were found between groups for the demographic factors of parents’ age, sex, marital status, employment status, or education level. To replicate the analyses used in Study 1, and because there was a significant effect of parents’ sex on one of the three outcome variables (parent’s emotional intensity during the incident), parents’ sex was held as a covariate during analyses. Controlling for parents’ sex did not change the significance of the results.

To examine the impact of the partner’s presence on parents’ emotional intensity, parental emotion regulation, and parents’ interpretation of their children’s emotion regulation, a mixed linear model was conducted for repeated measures using the partner’s presence as the independent variable and parents’ ratings of their own emotional intensity, parental emotion regulation, and child emotion regulation as the dependent variables. As each participant provided multiple responses over several weeks, a nested random structure was used with a by-week random variable nested within a by-individual random variable. This nested model failed to converge for the analysis of the child’s emotion regulation. Therefore, we separated the two random effects, thus creating a by-participant and by-week random effect.

**Figure 2:**
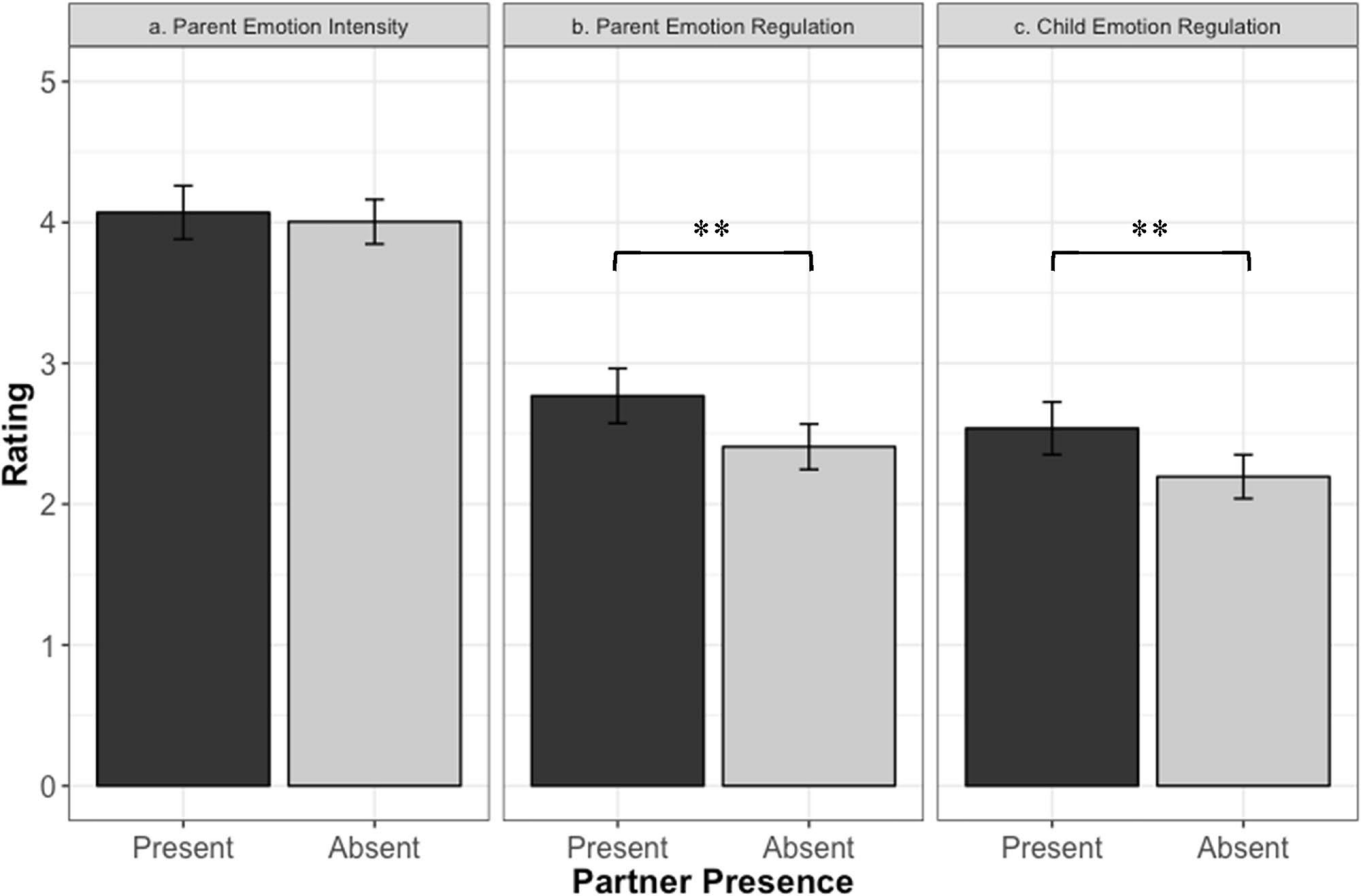
Parents’ ratings of their own and their child’s reactions to the emotional charged interaction with and without their partner present in Study 2. *Note* (**a**) Parents’ ratings of the intensity of their own emotions with and without their partner present (**b**) Parents’ ratings of their own emotion regulation with and without their partner present (**c**) Parents’ ratings of their child’s emotion regulation with and without their partner present. Error bars represent 95% confidence interval.

Results suggested that there was no significant difference in the level of parents’ emotional intensity when their partners were present (M = 4.07; SD = 1.04) compared to when their partners were absent (M = 4.0; SD = 1.10; b = − 0.066 [− 0.29, 0.16], SE = 0.11, t(292) = − 0.58, *p* = 0.564, Fig. 2a) during the emotionally charged interaction with their child. However, we found that when the parents’ partners were present, parents were better able to regulate their own emotions (M = 2.77; SD = 1.07) compared to when their partners were absent (M = 2.41; SD = 1.12; b = 0.36 [0.12, 0.61], SE = 0.13, t(287) = 2.89, *p* = 0.0042, Fig. 2b). Finally, we found that parents’ ratings of their child’s ability to regulate their emotions were significantly higher when the parent’s partner was present (M = 2.54; SD = 1.02) compared to when the partner was absent (M = 2.20; SD = 1.08; b = 0.34 [0.11, 0.58], SE = 0.12, t(300) = 2.85, *p* = 0.001, Fig. 2c). These results remained significant when controlling for the child’s age, severity of autism, and number of children in each family.

For cases in which the partner was present during the emotionally charged incident between parent and child, 50% had the partner involved in the incident. Yet the involvement of the partner had no significant effect on any of the three outcome variables: parents’ emotional intensity, parents’ ability to regulate their emotions, and children’s ability to regulate their emotions.

## Discussion

In two separate studies we examined the effect of the presence of parents’ romantic partners during emotionally charged interactions with their children on three separate outcomes: the parents’ assessment of their child’s ability to regulate their emotions, the parents’ ability to regulate their emotions, and the intensity of the parents’ emotions. As hypothesized, our studies demonstrated the positive effects of the partner’s presence on emotion regulation. Parents of both groups perceived their children as more regulated when their partner was present, and parents of autistic children perceived themselves as more regulated when their partner was present. There was no significant difference in the parents’ level of emotional intensity when their partners were present compared to when they were absent during the emotionally charged interaction with their child in either study.

### The effects of the partner’s presence on the child’s emotion regulation

In terms of the child’s emotion regulation abilities, parents perceived a significantly higher ability when their partner was present in both studies. These findings are consistent with prior research indicating the positive impact of social presence on emotional regulation^1–3,5^. One plausible explanation could be that the parent becomes more attuned to the child’s needs and can provide better emotional support for their regulation when their romantic partner is present. It is possible that the parent feels supported by his or her partner’s presence and therefore experiences greater parental competence, which in turn may contribute to the parent’s own regulation, consequently influencing the child’s regulation positively. This assertion aligns with previous studies suggesting that a regulated parent tends to result in a regulated child.

Another potential explanation could be attributed to the notion that in emotional interactions with the child, the presence of both parents may create a more relaxed and secure environment for the child compared to interactions involving only one parent. If the presence of both parents indeed has a calming effect on the child, it could elucidate the parents’ positive evaluation of the child’s emotion regulation when the partner is present compared to when they are absent. Understanding the impact of partner presence on children’s emotion regulation skills holds particular significance as it offers valuable insights for parents seeking to optimize their interactions during emotionally charged moments with their children. Recognizing the influence of both parents being present in such situations is crucial for potentially enhancing children’s emotion regulation abilities.

### The effects of the partner’s presence on the parent’s emotion regulation

As hypothesized, parents of autistic children demonstrated enhanced emotional regulation when their partners were present compared to when they were absent. Interestingly, among parents of non-autistic children, we did not observe a similar positive effect of partner presence on emotional regulation. In other words, these parents did not report improved emotional regulation when their partners were present versus absent. This deviation from our expectation contrasts with findings from previous studies indicating the positive effects of partner presence^12,13^. Additionally, research suggests that romantic relationships positively influence emotion regulation^35^.

One possible explanation for this disparity could be the heightened stress experienced by parents of autistic children compared to those of typically developing children. This heightened stress may amplify the impact of the partner’s presence among parents of autistic children. Collaborative efforts between both parents have been shown to be effective in managing challenging child behaviors, underscoring the importance of teamwork in navigating difficult parent–child interactions^36^. Moreover, a study by May et al.^37^ found that parents whose children receive a diagnosis of ASD place greater emphasis on developing and nurturing an effective co-parenting relationship.

Conversely, it’s plausible that parents of typically development children do not experience this effect because the interactions they have with their children are not perceived as stressful. Therefore, the presence of the partner may have less influence since the need for regulation is minimal. This aligns with studies demonstrating heightened emotion regulation in the presence of others during stressful situations^19,38^, suggesting that if the interaction is not perceived as stressful, this effect may not manifest.

### The effect of the partner’s presence on the parent’s emotional intensity

Contrary to our initial hypothesis, in both studies, we did not observe any variation in the intensity of emotions experienced by parents when their partners were present or absent. This unexpected finding prompts the question: why did we not observe this effect on parents’ emotional intensity? One plausible explanation could be that while the parent’s emotional intensity remains consistent regardless of the partner’s presence, his or her perceived ability to regulate his or her child’s emotions may fluctuate. In other words, in these studies, the partner may not have served as a buffer against the parent’s experience of overwhelming emotions. However, the partner’s presence still might have been beneficial in supporting the child’s emotion regulation process. This suggests that while the partner may not directly influence the parent’s emotional intensity, he or she may play a significant role in facilitating the child’s emotional regulation.

## Conclusion

While the current findings provide valuable insights into the influence of partner presence on parent and child emotion regulation abilities, these studies are not without limitations and warrant attention in future research endeavors. First, a notable limitation stems from the correlational nature of both studies. Rather than manipulating partner presence, we merely observed it. This limitation restricts our ability to establish causal relationships, as interactions when the partner is present may systematically differ from those when the partner is absent. To address this limitation, future research should employ the experimental manipulations of partner presence. Second, the sole reliance on parents’ self-report data presents another limitation. Utilizing alternative measures such as physical indicators or behavioral coding in future studies could provide a more comprehensive understanding of the phenomenon of partner presence. A third limitation lies in the exclusive focus on interactions that only induce negative emotions, primarily anger-inducing situations. While negative emotions are indeed integral to parenting interactions and child rearing, exploring the effects of partner presence on a wider range of emotions is key. Different emotions may elicit distinct effects pertaining to the presence of a partner, necessitating further investigation. Furthermore, while studying naturally occurring situations offers valuable insights beyond laboratory settings, it also introduces limitations. The inability to isolate the mechanism under study from other variables impedes our ability to draw clear conclusions. Lastly, the omission of assessing the quality of the relationship between romantic partners in this study poses another limitation. Given its significance in past research, future studies should incorporate assessments of relationship quality to better understand its role in the effects of the partner’s presence on parent or child emotion regulation abilities. In conclusion, the multitude of variables at play in natural situations, coupled with the inability to measure or control for all of them, underscores the need for cautious interpretation of findings and emphasizes the importance of addressing these limitations in future research endeavors.

### Supplementary Information

Supplementary Information.

## Data Availability

All data generated or analyzed during this study are included in this published article and its supplementary information files.
